# Healthcare providers’ perspectives of the supportive care needs of women with advanced breast cancer in Ghana

**DOI:** 10.1186/s12905-022-01931-7

**Published:** 2022-08-18

**Authors:** Cynthia Pomaa Akuoko, Shirley Chambers, Patsy Yates

**Affiliations:** 1 Department of Nursing and Midwifery, Christian Service University College, Kumasi, Ashanti Region Ghana; 2 Cancer and Palliative Care Outcomes Centre, School of Nursing, Queensland University of Technology, Brisbane, QLD Australia; 3 Centre for Healthcare Transformation, Faculty of Health, Queensland University of Technology, Brisbane, QLD Australia

**Keywords:** Advanced breast cancer, Healthcare providers, Supportive care needs, Ghana

## Abstract

**Background:**

The study sought to understand the supportive care needs of women with advanced breast cancer from the perspectives of healthcare professionals (HCPs) and key informants of charitable/non-governmental organisations (NGOs), that provide supportive care services to women with advanced breast cancer, in Ghana.

**Methods:**

A qualitative descriptive approach was employed via one-to-one semi-structured interviews with 13 HCPs and key informants of charitable/NGOs in Ghana that provide supportive care services to women with advanced breast cancer. The study was underpinned by Bradshaw’s taxonomy of social needs and Fitch’s supportive care framework. The data were analysed using a deductive content analysis approach.

**Results:**

Healthcare providers and key informants perceived that women with advanced breast cancer in Ghana have numerous and complex supportive care needs in key areas that align with Fitch’s supportive care framework, including informational, psychological, emotional, physical, practical, social, sexuality and spiritual needs.

**Conclusion:**

Participants perceived that women who have advanced breast cancer in Ghana require ongoing information about their condition, treatments and related effects, as well as spiritual support and guidance particularly due to the fatalistic beliefs they often associate with the condition. Tailored supportive care interventions and services, which address the unique sociocultural circumstances for this cohort, are required. Additional research is needed to explore how multidisciplinary teams can work collaboratively to provide comprehensive support to women in addressing their needs.

**Supplementary Information:**

The online version contains supplementary material available at 10.1186/s12905-022-01931-7.

## Background

In 2020, breast cancer was the most frequently diagnosed cancer in women worldwide [1] and accounted for 29.5% of cancer incidence and 22.1% of cancer deaths in Africa [2]. In Ghana, breast cancer accounted for 31.8% of cancer incidence [2]. It is more common to be diagnosed with advanced breast cancer in African countries, when compared to Western countries, [3, 4] as evidenced by around 70% of women being initially diagnosed in Ghana with advanced breast cancer breast cancer [5]. Contributing factors for this phenomenon include poor recognition and appraisal of breast cancer symptoms, fear and denial, sociocultural beliefs, financial and health system impediments, and the absence of screening programs [6–9].

Women with advanced breast cancer have been found to experience multiple and complex supportive care needs across seven domains [10], more specifically informational (e.g., underserved with available information), psychological (e.g., shame, guilt, depression), emotional (e.g., neglect), social (e.g., withdrawal, isolation), physical (e.g., pain, fatigue), practical (e.g., unemployment, decline in income) and sexuality related needs (reduced libido, intimacy issues) arising from their illness and treatments [11–16]. Recognition and validation of patients’ needs enhances open dialogue between patients and healthcare professionals (HCPs) which leads to referrals to appropriate services [17].

The negative impact of cancer and its treatment on patients has been acknowledged by HCPs worldwide [10]. Earlier studies undertaken in Western developed countries have investigated HCPs’ perspectives on supportive care needs of patients with advanced prostate cancer [18], colorectal cancer [19], cancer patients during oncology treatment [20], and their caregivers [21], and on the prevalence, barriers, and management of psychosocial issues in cancer care [22]. To the best of our knowledge, no study has investigated the supportive care needs of women with advanced breast cancer from HCPs’ perspective in Ghana. Hence the aim of this study was to understand the supportive care needs of women with advanced breast cancer from the perspectives of HCPs and key informants, of charitable/non-governmental organisations (NGOs) in Ghana that provide services to women with advanced breast cancer, to help inform the planning and establishment of tailored interventions to address such needs.

The supportive care needs of women with advanced breast cancer in Ghana have recently been assessed, and reported, from the perspective of the women [11]. However, the needs of these women from the perspective of the HCPs and key informants of charitable/NGOs have not, to the best of our knowledge, been considered. Therefore, it remains unclear to what extent HCPs understand the supportive care and health service needs of women with advanced breast cancer in Ghana. The external assessment of the needs of patients by their HCPs is important in their management [23]. HCPs have acknowledged the impact of the effects of cancer and its treatments on patients [23–25] hence the need to provide better supportive care and health services to address the needs of these patients. Furthermore, insight into HCPs’ perspectives of the needs of this cohort may help ensure that appropriate and effective care and disease management care pathways and activities are instituted through appropriate referral process.

## Methods

### Conceptual framework

This qualitative exploratory study was part of a multiphase study that aimed to explore the supportive care and health service needs of women with advanced breast cancer. Bradshaw’s taxonomy of social need [26] and Fitch’s supportive care framework [10] underpin this study. Bradshaw classifies need into four interrelated categories: normative (desires defined by experts), felt (subjective desire for things), expressed (when such desires are turned into actions) and comparative (comparisons with others not in need) [26]. This paper reports patients’ needs according to the perception of experts (normative needs) [26], as conceptualised using Fitch’s [10] supportive care framework as an analytical framework, which includes psychological, emotional, physical, social, practical, informational, and spiritual domains.

### Participants and setting

Using a mixed, purposive sampling strategy, which employed both a nomination selection strategy [27] and maximum variation sampling [28], HCPs and key informants of charitable/NGOs (from here referred to as experts) who support women with breast cancer in Ghana were recruited. The participants were selected from the oncology directorate/unit of Komfo Anokye Teaching Hospital and Peace and Love Hospital and the NGOs of Breast Care International and Breasted One Foundation in Ghana.

### Procedures

Recruitment site executives identified potential experts and provided them with study information. Interested experts were contacted by the researcher, upon their permission, by email, phone and in person to explain the study further and to answer questions. Each of these experts received an information leaflet. Written consent was received prior to interview.

### Data collection strategy

One-to-one, semi-structured interviews were conducted by phone or face-to-face at the expert’s workplace in 2019; duration average was 38 min (range 17–67 min). To ensure all relevant and related concepts were explored, a purpose-specific interview guide (Additional file 1) was piloted with an oncology clinician and a key informant in Ghana from a similar NGO. Topics covered the supportive care needs of women with advance breast cancer and the existing services available to address such needs. Each interview session was digitally recorded with participant consent.

### Data analysis

The study was conducted following the COREQ criteria [29]. Interview recordings were transcribed verbatim. An established deductive/directed content analysis method was utilized [30]. The lead author read the transcripts multiple times to gain understanding of the data. Key concepts, based on Fitch’s [10] supportive care framework, were used to develop the initial coding scheme. The categorisation matrix comprised subcategories and broader tentative categories of data. The assignment of codes to categories was randomly assessed by author 2; changes were made as appropriate upon agreement. Categories were continuously refined until all statements/responses were appropriately coded. All authors reviewed the categorisation matrix to establish congruence of understanding and to reconcile ambiguous interpretations [31].

### Study trustworthiness

Study credibility was strengthened by establishing rapport with participants. The first author is a nurse and had previously cared for women with advanced breast cancer. Purposive sampling, and the collection and analysis of thick and rich descriptive data, boosted transferability of results. Dependability of study results was increased as the study protocol was reviewed by independent researchers, by using data analysis processes that involved more than one researcher, and by peer debriefings during analysis processes. An audit trail of research activities enhanced study confirmability [32].

## Results

### Sample characteristics

All 13 eligible potential experts identified consented to participate. Their median age was 40 years (range 25–61 years) and most were female (61.5%). Table 1 summarises the characteristics of the experts.

**Table 1:** Characteristics of the expert participants

Characteristics	Participants
	N = 13
Age	*Median (range) years*
	40.0 (25–61)
Number of years worked with women with Breast Cancer	9.0 (3–14)

	*n (%)*
*Sex*	
Male	5 (38.5)
Female	8 (61.5)
*Discipline*	
Medicine	4 (30.8)
Nursing	4 (30.8)
Counselling	2 (15.4)
Dietician	1 (7.7)
Physician assistant	1 (7.7)
Social worker	1 (7.7)

### Supportive care needs

Data were categorised into Fitch’s [10] original seven domains of supportive care needs. Upon analysis, an additional domain—sexuality needs—was created. Sexuality needs, as a domain, were originally embedded within the psychological domain of Fitch’s framework. However, these data were grouped in a separate category due to the extent and depth of findings relating to sexuality needs. Results are reported against first and second level categories that align with the supportive care domains (details in Table 2).

**Table 2:** Supportive care needs as perceived by the experts

Supportive care domains	1st level categories of need	2nd level categories of need
Informational needs	What cancer means	Understand the disease process
	Cause/risk factors	
	Dispel misconceptions	
	Being proactive about information seeking	
	Treatment options	Understand service/treatment issues/care processes
	Prognosis/outcomes	
	Adherence to treatment	
	Side effects/complications	
	Cost of treatment	
	Existing support services	
	Ongoing information	
	Oral information	Sources of information
	Written information	
	Audio visual (videos)	
	Internet	
	Doctors/nurses	
	Allied health professionals	
	Survivors of breast cancer	
Psychological needs	Self-esteem issues	Keep a positive outlook
	Body image issues	
	Depression	Psychological support
	Anxiety	
	Fear	
Emotional needs	Shame	Manage feelings
	Need to be loved	
	Need to be heard	
	Empathy	Moral support
	Reassurance	
	Family support	
	Peer support from cancer survivors	
Physical needs	Symptom relief	Physical comfort
	Relief of side effects/ complications	
	Comprehensive treatment preparation	
	Nutritional support	Physical support
	Exercise advice	
Practical needs	Assistance with usual daily tasks	Practical support
	Financial assistance	
	Employment assistance	
	Accompaniment to appointments	
	Childcare support	
	Accommodation support	
Social needs	Social inclusion	Acceptance
	Openly discuss condition	
	Community support	
Sexuality needs	Spousal support	Counselling on sexual relations
	Sexual counselling for low libido	
	Advice on intimacy issues	
	Fertility advice	Physiological advice
	Advice on menstruation/ menopause issues	
Spiritual needs	Seek meaning	Find meaning and purpose
	Existential understanding	
	Value life	
	Accept reality	
	Spiritual support	Find peace
	Spiritual guidance	
	Strengthen spiritual beliefs	
	Hopefulness	

### Informational needs

The second level categories identified include ‘understand the disease process’, ‘understand service/treatment issues/care processes’, and ‘sources of information’.

#### Understand the disease process

The provision of information to women to help them to understand what cancer means needs to be conveyed in their own dialect and in easy-to-understand terminology. According to the experts, the cause/risk factors related to breast cancer should be part of the information provided to these women, as they tend to have different views of what causes cancer. They described some of the misconceptions/myths/fallacies about breast cancer that need to be dispelled, such as the cancer being caused by spiritual forces. These beliefs tend to be strong, particularly when there is no family history of breast cancer. They also noted that the women need to be proactive about seeking information about their condition. (Table 3, quotes 1–4).

**Table 3:** Informational need; categories identified and related quotes from the experts’ perception of the supportive care needs of women with advanced breast cancer

2nd level categories of needs	1st level categories of needs	Examples of experts’ narratives	Reference number of quotes
*Informational needs*			
Understand the disease process	What cancer means	*I think the patient must know the kind of diagnosis she has. So, the name of the disease in the local language and the medical language should be given to the patient, and in as much* [detail] *as possible… (Expert 08)*	1
	Cause/risk factors	*You know cancer in our parts of the world we have different views about what it really means. So, they need to know what it is about because there's one question that most people normally ask. How did I acquire it? Is there something I can do? Or something I did wrong. So, there are a lot of questions that we need to help these people* [women] *to find answers to.* (Expert 01)	2
	Dispel misconceptions	*People* [women] *still have that mentality that they are spiritual* [the cancer is caused by spiritual forces] *especially when there's no history in the family.* (Expert 01)	3
	Being proactive about information seeking	*Most of the time what I have observed is that they have that problem talking to doctors about the disease condition. Some of them tend to see doctors as super humans and so they think that the doctor is all knowing, and the doctor has rights over their disease condition or has rights over them. And so, whatever the doctor says they just take it without probing, without asking further questions. But when they come out from the doctor’s consulting room that is when you the nurse, they come to you and they try to engage you.* (Expert 06)	4
Understand service/treatment issues/care processes	Treatment options	*You need to let the patient know what is available in terms of treatment. No matter the stage…something that can be done for the patient. So, I think in terms of information, that should be made available to the patient.* (Expert 08)	5
	Prognosis/outcomes	*And then apart from the diagnosis the patient deserves to know the prognosis.* (Expert 08)	6
	Adherence to treatment	*They must always be punctual at the hospital, take their medications well* [as prescribed]. (Expert 12)	7
	Side effects/complications	*How to cope with the treatment whether it’s side effects of the chemotherapy, radiotherapy and all that. And how to cope with I mean any other thing that will come or any other complication they might develop after the treatment, lymphedema and all those things* (Expert 02)	8
	Cost of treatment	*We also bring them to appreciate gradually the cost that maybe involved in the investigations and the treatments that they are going to encounter.* (Expert 07)	9
	Existing support services	*The existing services around the communities one thing is most patients may not be aware there is a service that can help them with their situation that they find themselves. Most of them are not aware and as a matter of fact they are not using it* [accessing the services]*.* (Expert 01)	10
	Ongoing information	*As for the information they need it all the time. Each time they visit you still need to hammer on some of the points that maybe the patient has gone through.* (Expert 13)	11
Sources of information	Oral information	*“It has to be verbal…verbal, I think sinks* [in] *more, especially in our setting where most of our patients are not educated.”* (Expert 08)	12
	Written information	*It could be in leaflet form.* (Expert 03)	13
	Audio visual (videos)	*Other source like…something in audio visual about the conditions for them* (Expert 03)	14
	Internet	*Those who are enlighten can also go online to read about it.* (Expert 03)	15
	Doctors/nurses	*Anybody who sees the patient* [can provide information to the women] *provided the person is well resourced to give that information.* (Expert 02)	16
	Allied health professionals	*Here* [at recruitment site] *the counsellor and the psychologist talk to them.* (Expert 10)	17
	Survivors of breast cancer	*I think it* [information] *should be from survivors.* (Expert 11)	18

#### Understand service/treatment issues/care processes

Stepping the women through the various processes of their treating facilities, treatment issues, and/or the disease care pathways will ensure that they will be in a better position to cope with their condition, treatments, and related consequences. The women, too, need to be made aware of incurring high out-of-pocket expenses that are associated with receiving breast cancer treatments, as not all treatments and ongoing diagnostic tests are currently covered by the National Health Insurance Scheme (NHIS) in Ghana. Furthermore, some of the experts noted that these women need access to information about existing support and health services (Table 3, quotes 5–11).

#### Sources of information

The women could access breast cancer information in writing, via the internet, and in audio-visual format, however, most experts noted that oral communication of the relevant information is best for this cohort. Some experts suggested that the information be provided by members of the multidisciplinary team, including social workers and psychologists, adding a caveat that these personnel must have a good understanding of breast cancer and related topics to well equip the women for their cancer trajectory (Table 3, quotes 12–18).

### Psychological needs

‘Keep a positive outlook’ and ‘psychological support’ were the second level categories that were identified by the experts.

#### Keep a positive outlook

Fungating lesions experienced by women with advanced breast cancer, due to ulcerations and necrosis of the breast, may exude a foul odour which can lead the women to withdraw. Side effects from treatments, such as loss of a breast, a wound, and/or a scar from radiotherapy can interfere with the choice and styles of clothes they wish to wear. These issues aside, according to the experts, the most pressing problem experienced by these women is the importance that society places on women’s breasts as the most important external identification of femininity. As these issues can negatively impact women’s self-esteem, these issues need to be acknowledged and address to assist them to keep a positive outlook. (Table 4, quote 1–2).

**Table 4:** Psychological and Emotional needs; categories identified and related quotes from the experts’ perception of the supportive care needs of women with advanced breast cancer

2nd level categories of needs	1st level categories of needs	Examples of experts’ narratives	Reference number of quotes
*Psychological needs*			
Keep a positive outlook	Self-esteem issues	*Most of them have low self-esteem because you see advanced breast cancer doesn’t only have the name advanced, it has certain things that goes with it that is why it is described as advanced.* [In] s*ome the breast become ulcerated. Now the offense that is emanating from this breast is sometime so bad that these women find it difficult especially when they come and they have to sit at the reception to wait for their turn to be seen by the doctor, they feel very uncomfortable sitting with other woman because everybody tend to realise that there is some kind of bad odour coming out from you and therefore they are left alone on that seat on which they sit. So, it really affects their self-esteem.* (Expert 06)	1
	Body image issues	*The fact that somebody is having a wound, it has a lot* [sic] *on the self-esteem. The person cannot wear the type of clothes that she is supposed to wear. The person has had mastectomy, you know, and she’s thinking that breast is gone, and you know how women and society view breasts as, I mean it’s your whole epitome of being a woman. So, it affects the woman’s self-esteem in a negative way.* (Expert 04)	2
Psychological support	Depression	*Psychologically, this patient once has* [sic] *been diagnosed with advanced disease is destabilised. Because of the conception we have that once you have cancer you will die, this patient is demoralised. So, psychologically she is not sound again because she is going to think about what happened to her.* (Expert 06)	3
	Anxiety	*Women pride in the size of their breast, the shape of their breast, even some men choose their wives base on the size of their breast. So if the woman knows the husband developed interest in her because of the breast and then one day she develops a disease which has to take away the breast then it means as soon as that thing is mentioned the woman thinks now I’m going to lose my husband because she associate the husband to the breast.* (Expert 08)	4
	Fear	*The psychological needs basically revolve around how to cope with the disease because like the fear of the unknown, they don’t know what will happen and that bothers them.* (Expert 02)	5
*Emotional needs*			
Manage feelings	Shame	*People* [the women] *will not even want people to get to know that they've even been diagnosed of cancer, no matter how old they are, even the elderly ones wouldn't want people to know or come to the knowledge that they've been diagnosed of cancer.* (Expert 01)	6
	Need to be loved	*Emotionally the person needs to be loved and the person needs to feel that although I am having one breast, although one arm is swollen my husband still loves me.* (Expert 04) *If you are in this situation you need more comfort, you need more love, care so that you feel like you are a person than being rejected.* (Expert 12)	7
	Need to be heard	*As much as possible especially in advanced disease or metastatic disease I think we should listen to the patient. It shouldn’t just be about what you want to do for the patient because in metastatic setting you know that most of the things or what you are doing for the patient is palliative. It’s not meant to cure the patient, it’s to make the patient have a better quality of life and you know you can’t decide what quality of life is for the patient, the patient must decide. So, we need to listen to the patient. Let the patient tells you that this and that she has to do, there is a ritual she has to do, some prayers she has to say, some people she has to see, you need to factor all that in your treatment framework. So that in the end you are not just doing what you think is going to help the patient but then the patient is also involve in that process. I think it works better that way.* (Expert 08)	8
Moral support	Empathy	*Contact, that is what they need. They need empathy, they need someone to share with them their pain*. (Expert 05)	9
	Reassurance	*Emotionally they are equally affected, and so they need someone who could or who is always there to support them emotionally, not only the healthcare givers, but* [I’m] *talking about the family, the carers, because some of these women will come and they come alone.* (Expert 06)	10
	Family support	*There should be a lot of support from the family members. They have to give them all the moral support that they will need to go on with this treatment because the person after being diagnosed is even down, the person is emotionally weak so if those around her rejects her it even worsens the situation and it speeds up the death process.* (Expert 01)	11
	Peer support from cancer survivors	*So, I believe that there must be people who will be able to give that presence that support every now and then besides family. I will also advocate that we tend to look forward having kind of support groups, formation of support groups so that those who are living with it will be able to share their experiences to help others to live with it.* (Expert 07)	12

#### Psychological support

The experts noted that women feel a sense of depression due to the loss/mutilation of a breast as feelings of reduced femineity ensue. Fatalistic ideas related to a cancer diagnosis in the Ghanaian context further causes fear. Experts also noted that the women need help to work through their psychological issues such as anxiety, uncertainty related to their condition, fear for their future and the unknown, and fear of recurrence and death (Table 4, quotes 3–5).

### Emotional needs

The second level categories that explain the experts’ perceptions of the *Emotional needs* of this cohort included ‘manage feelings’ and ‘moral support’.

#### Manage feelings

Women with advanced breast cancer experience shame due to the cancer diagnosis, and according to the experts, these issues need to be addressed to support their emotional stability. The experts noted that women are often abandoned, which has negative consequences. In their opinion, these women need more comfort and love than was needed prior to their diagnosis, and as such, need to feel appreciated and heard (Table 4, quotes 6–8).

#### Moral support

The experts noted that these women need empathy and constant reassurance from their families and friends, HCPs, and from other women in similar situations. Interacting with other women who have thus far survived having breast cancer can provide women with a sense of belonging and moral support as they share stories of their disease trajectory. (Table 4, quotes 9–12).

### Physical needs

The second level categories that derive from this domain include ‘physical comfort’ and ‘physical support’.

#### Physical comfort

According to the experts addressing physical symptoms, such as pain, fatigue, nausea/vomiting, ulceration of the breast, and offensive odour, is paramount to help women cope with their condition. Furthermore, the experts believe the women need comprehensive preparation to adapt well to and cope with life before and after treatment. (Table 5, quotes 1–3).

**Table 5:** Physical and Practical needs; categories identified and related quotes from the experts’ perception of the supportive care needs of women with advanced breast cancer

2nd level categories of needs	1st level categories of needs	Examples of experts’ narratives	Reference number of quotes
*Physical needs*			
Physical comfort	Symptoms relief	*They go through a lot of pain…and loss of appetite…weight lost…nausea…these are some of the side effects of the treatment.* (Expert 01) *Fatigue is a side effect of the treatment, so they go through that. Lymphedema is also one of them because here they present with an advanced disease.* (Expert 02) *They also experience darken skin and then alopecia.* (Expert 03) *They have a wound…they have fungating breast…the odour is bad.* (Expert 04) *The acute side effects* [include] *vomiting sometimes even bleeding,* [and] *diarrhoea.* (Expert 08)	1
	Relief of side effects/complications	*So, what happens is that before we start treatment, we counsel the patients. We tell them what will happen to them…once we tell them the side effects of the treatments, we also explain to them how to manage it at home.* (Expert 04)	2
	Comprehensive treatment preparation	*I don’t think they are given any proper preparation…I went to a particular ward* [site] *and most of them didn’t even know much about the condition. So, I don’t think that it is properly done.* (Expert 11)	3
Physical support	Nutritional support	*Other physical needs [that] you can think about is maybe how well nourish*[ed] *they are. I mean, that has to do with nutrition and that borders on counselling, and then maybe bringing in the nutritionist, because often they live under the impression certain foods can even cause cancer.”* (Expert 09)	4
	Exercise advice	*And for some of them, like the postmenopausal women, or for some of the drugs it can make their bones lighter and more liable to fracture. So there too we need to advise some kind of exercise, [though] not [a] very vigorous one.* (Expert 09)	5
*Practical needs*			
Practical support	Assistance with usual daily tasks	*Functionality, you know breast cancer usually comes with lymphedema and the fore limb at the side of where the cancerous lesion is it’s usually affected and definitely cannot be used for daily activities, washing, and they become fatigued…also anaemia. It brings down their functionality, they are not able to function as before.”* (Expert 05)	6
	Financial assistance	*It has a great impact on their finances, seriously, because the treatment is not free even though the government pays for some of the treatment, it’s still not free. It’s very expensive. It is three years down we don’t have most of the drugs coming in, okay. So, they have to go out and buy* [them] *and sometimes they skip their treatment because they don’t have money to buy* [them]*. So, it’s a big toll on their finances. Sometimes they have to depend on friends, families to donate. Sometimes* [they have to go] *to the extent of selling their properties.* (Expert 06)	7
	Employment assistance	*For those who are in government, and civil and public, they don’t have issues with employment, but those who are working in private firms and their own jobs and* [as] *entrepreneurs, they have issues with the employment. The entrepreneurs, they just leave their job and come, but then the job is also suffering…the money that she is using to purchase the drugs is coming from that same job.* (Expert 04)	8
	Accompaniment to appointments	*I think most of them they need the support of the family, they want their relatives to be around. That is the practical needs. So, they want their family members to follow them to the hospital, they want if they are married, they want their husbands to be supportive that is what they want *(Expert 03)	9
	Childcare support	*Especially for the ones, the young breast cancer patients who have kids, young kids. I mean some have toddlers and infants and those things, mm-hmm so they need to I mean care for those kids. And sometimes it’s difficult for them so they need help* (Expert 04)	10
	Accommodation support	*Those who come from outside Kumasi who have to travel some distance, in the first place they don’t even have money for their treatment, let alone money to rent an apartment or get a decent sleeping place, they come and have to sleep at the open space…So, these women need a place to stay.* (Expert 06)	11

#### Physical support

Some of the experts noted that the women need physical support in relation to nutrition and exercise advice due to the significant impact that the disease progression, and its management, can have on their eating patterns and bone density. (Table 5, quote 4–5).

### Practical needs

The second level category of this domain is ‘practical support’.

#### Practical support

The women require practical assistance with daily tasks, such as washing and cooking, due to the side-effects/complications of the disease and/or the treatments endured, which include becoming fatigued and anaemic, and from developing lymphedema.

In addition to high out-of-pocket costs associated with treatment, the nature of the disease and its treatments impacts the women’s physical strength, thereby limiting their work participation leaving them unemployed and financially drained. This results in the women needing financial and employment assistance for their business, and occupational counselling. Furthermore, as the women are physically weakened by their condition and treatment, they likely need to be accompanied to appointments and may need childcare support.

According to the experts, most women who access the two local oncology sites at Kumasi, live more than 300kms away. Some of these women are likely to need to be at the clinic a day prior to their appointments due to travel requirements. Consequently, these women need accommodation support to enable them to attend their scheduled appointments and to adhere to treatments (Table 5, quotes 6–11).

### Social needs

The second level category identified within the *Social needs* domain is ‘acceptance’.

#### Acceptance

The experts unanimously raised the issue of the stigma that these women endure from their families and society generally, explaining that as breast cancer in Ghanaian society is often branded as a condition contracted due to one’s sinful deeds or spiritual forces, these women tend to not openly discuss their diagnosis. Hence, community acceptance and support, which incorporates family and peer support, is needed by these women (Table 6, quotes 1–3).

**Table 6:** Social, Sexuality and Spiritual needs; categories identified and related quotes from the experts’ perception of the supportive care needs of women with advanced breast cancer

2nd level categories of needs	1st level categories of needs	Examples of experts’ narratives	Reference number of quotes
*Social needs*			
Acceptance	Social inclusion	*For some* [it] *is good, but others it’s a total neglect; they don’t accept them. The person is emotionally weak, so if those around her reject her, it even worsens the situation and it speeds up the death process.* (Expert 01) *Basically, what they want is social contact and people accepting them as they are.* (Expert 05)	1
	Openly discuss condition	*So, when it comes to families and friends knowing about diagnosis* [that] *is a no-go area.* (Expert 04) *When you have a disease, which is not curable traditionally they think you’ve contracted that because of something bad that you've done and for that matter people don’t want to associate with you.* (Expert 09)	2
	Community support	*And our society also does not place premium on giving societal support to these people* [women with breast cancer] *it is not done. We don’t even have a group for them.* (Expert 05)	3
*Sexuality needs*			
Counselling on sexual relations	Spousal support	*What I have noticed here over the years is that most of the women when they get breast cancer their spouses leave them. You have a few women who gets the spousal support. So, when it comes to the issue of sex, even for the woman to open herself up even after mastectomy even those who are in remission you know it is difficult.* (Expert 04)	4
	Sexual counselling for low libido	*You know, you need to be in some mode, state of mind to be able to approach the act of sex, to be able to enjoy the act of sex. So, I’m tempted to believe that it will be reduced so much than before and this whole psychology of sex….so, this whole concept of intimacy you know a number of things cut off intimacy,* [an] *example is the offensive smell.* (Expert 07) *When they go on the medications the chemotherapy especially it reduces their libido. So, sometimes when they are married people, they don’t feel like having sex with their partners.* (Expert 13)	5
	Advice on intimacy issues	[For] s*exual needs don’t look at just the coital aspect of it, it also comes with touching and all those things emotional connection of which they don’t get.* (Expert 05)	6
Physiological advice	Fertility advice	*And when it comes to fertility issues, once the person starts chemo, if the person is young, then they worry about their fertility issues. “Will I be able to have kids after this?” But if the person is old, they don’t really worry about fertility issues because they have already given birth. But for the young patients with advanced disease, sometimes they have issues with fertility*. (Expert 04)	7
	Advice on menstruation/menopause issues	*It really impacts on them, because for the young patients, they will kind of go into actual menopause and they experience some or most of the symptoms that go with postmenopausal women, and that is not comfortable.* (Expert 09)	8
*Spiritual needs*			
Find meaning and purpose	Seek meaning	*Sometimes most of the women will tell you that they don’t understand why they have this condition, and they hope that God will heal them. So, apart from the patient coming to this centre they also seek help from the churches and then other religious bodies.* (Expert 03)	9
	Existential understanding	*They feel that life is not even worth living with all these especially those who come with metastatic disease for which we say they cannot be cured. I mean they think it’s not worth living.* (Expert 09) *Often, they begin to ask themselves “why them”. I mean, for all the religious groups they belonged to and for all that they’ve been doing, why should such things happen to them… Sometimes they ask the doctor that, why could such a thing happen to them. “I’m a Christian, I’m a Muslim, I do things right. So how do these things happen?”.* (Expert 09)	10
	Value life	*I think for women with advanced cancer they really treasure life they really treasure every day that they live because you know for people who are up and about, who are not sick we think that it’s normal. I mean it's not a big deal. Then for somebody with an advanced breast cancer it like anything will happen in any moment. So, every single day they valued it.* (Expert 07)	11
	Accept reality	*So, why not spend much time with God and sometimes because of that they even forgo the treatment, you can start they just abandon the treatment go to their pastors to pray, and by the time they come back, it’s even much worse than before. So, at that point that is how most of them behave*. (Expert 02)	12
Find peace	Spiritual support	*They feel since it’s spiritual, it has to be handled in the spiritual manner. So, instead of them coming to the hospital for medications, it becomes something else. We are competing with the pastors. We are competing with the herbalists. So, how they want to present the case to them, the spiritual side of the whole thing and how we also want to portray the medical side of the whole thing. So, now we tell them when you go home—pray. Prayer is good. We all will be praying, but when it’s time for your medication, please come. To some extent we are helping these patients to know “Oh, so it’s not all that bad” and to some extent it gives them a very big relieve* [sic] *because they feel they can do both.* (Expert 01)	13
	Spiritual guidance	*For our people for the patient, women with advanced breast cancer they really need to be I mean guided spiritually* (Expert 04)	14
	Strengthen spiritual beliefs	*Once they are diagnosed, what I have realised is that most of them, spiritually, they become stronger. They try to get closer to God because then they know its advanced disease, they may just die at any time. So, why not spend much time with God?* (Expert 02)	15
	Hopefulness	*I always give a scenario of separate group of people. One group once they are told they have advanced disease okay then the hopes and everything is shattered. However, the other group of people are those ones who irrespective of being diagnosed of advanced disease will fight to live and you always see it that such people are always fighting and anything they are asked to do they strive to do it because they have that at the back of their mind that they want to survive. *(Expert 06)	16

### Sexuality needs

The identified second level categories of this domain were ‘counselling on sexual relations’ and ‘physiological advice’.

#### Counselling on sexual relations

According to the experts, the women encounter sexual problems, such as reduced libido, which impacts their intimate relationships. In their opinion, women with advanced breast cancer need spousal support, however many women do not receive such support (Table 6, quotes 4–6).

#### Physiological advice

The experts claimed that younger women with advanced breast cancer, in particular, need physiological advice from HCPs in relation to being propelled into early and often abrupt menopause, and/or experiencing the temporary cessation of their menstrual cycle, due to the cancer treatments, and consequential fertility concerns (Table 6, quote 7–8).

### Spiritual needs

The second level categories related to this domain are ‘find meaning and purpose’ and ‘find peace’.

#### Find meaning and purpose

Women with advanced breast cancer pursue meaning in their diagnosis. One way they navigate their diagnosis, according to the experts, is by seeking help from their religious leaders. The need for existential understanding, that is to find answers to questions that may lead to understanding the purpose of God in their lives, to be more accepting of their situation, and to truly value their remaining days, is often expressed by these women (Table 6, quotes 9–12).

#### Find peace

The experts reported that many Ghanaian women believe in the spiritual aspects of the disease and often tend to seek spiritual peace more than the physiological benefits of treatments. Paying attention to their spiritual concerns, by supporting, guiding and affirming that they can seek both spiritual and physiological benefits while being treated for the disease, may help the women to find peace (Table 6, quotes 13–16).

## Discussion

To the best of the authors’ knowledge this is one of few studies to explore the needs of women with advanced breast cancer in Ghana from the perspectives of relevant experts. Although the findings of this study are consistent with those of other studies which indicate that women with advanced breast cancer have numerous supportive care needs [11–16], this study provides additional insights from these experts’ perspectives about the plight of women in Ghana.

Similar to other studies of women with advanced breast cancer [12–14], informational needs are an important priority for women in Ghana. These women require ongoing information about their condition, treatment modalities, expected side effects/complications and prognosis/outcomes, as well as the expected costs involved with investigations and treatments, and the need to comply with disease management instructions. Participants noted that women can have many beliefs and misconceptions that need to be understood, considered and explored to encourage adherence to treatments [33]. Such information can be provided in a range of formats, such as in written or audio-visual format, according to their needs and in their spoken language [34].

Psychological issues raised by the experts focused on loss of control in relation to the women’s depression, anxiety, fear, altered body image and low self-esteem. These findings are similar to those reported by women with advanced breast cancer in European countries, Ghana, Iran and Canada [14, 35–37]. The breast plays an important role in the definition of womanhood, attraction and beauty therefore losing a breast can psychologically impacts the woman’s self-worth [38, 39]. Gaining personal control by changing one’s perspective of the condition and looking for positive opportunities in face of their circumstances proved to be helpful according to the experts.

Experts in this study noted that women with advanced breast cancer can feel embarrassed, unloved and feel that their concerns and feelings are not considered, similar to findings from a global study of women with advanced breast cancer [14]. Support, provided by their treating team, family members, friends and breast cancer survivors, is required to help them manage such feelings to facilitate their emotional stability as they navigate their cancer trajectory [40].

Symptoms, side effects and complications experienced by women were also noted by the experts as requiring prompt and effective management, including nutrition and exercise advice. Not all the experts agreed that the women with advanced breast cancer in Ghana are prepared in these areas, highlighting that more comprehensive supportive interventions before and during treatment are warranted [41].

The practical needs raised by the experts are similar to those raised by the women with advanced breast cancer in a global population-based representative study [14] and by women living with breast cancer in rural Canada [37]. Managing activities of daily living when faced with advanced illness is problematic [42]. Employment issues together with the out-of-pocket costs related to cancer treatments and investigations contribute to the women’s financial difficulties. Accommodation and transport are issues for many Ghanaian women who live far from treatment sites. These issues require intervention at the policy and community level to provide financial support and community development programs.

Women with advanced breast cancer need to feel accepted by both their families and their communities and in a country, such as Ghana, this is vital where there is no advanced breast cancer peer support groups [43]. Similar to the views expressed by the experts in this study, Cardoso and colleagues [14] reported that many women perceive that society views women negatively once diagnosed with advanced breast cancer, leading to a sense of rejection. The experts noted that a diagnosis of advanced breast cancer further puts a strain on the women’s relationships with others and, by default, they do not receive the social support they need. The experts in this study suggested social support be provided by the treating team as it may be the only social support the women receive.

According to Barsotti and colleagues [38], a range of sociocultural factors can negatively impact women diagnosed with breast cancer, including a traditional emphasis on women’s sexual expression being contained within marriage, prioritisation of male sexual pleasure, and notions of the female breasts and their sexual attractiveness. These factors can prevent the women from seeking the support needed to address sexual issues, such as reduced libido and intimacy problems [38]. Moreover, a women’s relationship with her spouse/partner can be negatively affected due to changes in mood or negative perceptions about themselves [14]. Women often link their appearance, weight, and body image to their perceptions of attractiveness which can impact sexual relationships after a diagnosis of, and treatment for, ABC [39, 44]. At a time when women with advanced breast cancer need support from their intimate partners, experts in this study observed that women can feel abandoned. This differs somewhat from findings of a Brazilian study which reported that women’s partners supported them despite the intense emotional stressors they experienced [38]. Fertility and menstruation/menopause issues were also concerns for women, as noted in other studies [45]. The provision of timely and appropriate advice from qualified HCPs is needed to address these sexual and fertility concerns to support the women.

The experts in this study also highlighted that women with advanced breast cancer can struggle with existential concerns and have unrealistic expectations about life post-diagnosis. Spiritual needs have been reported by cancer patients in other studies [46, 47] including a study in Iran [48]. Spiritual support and guidance is therefore an essential component of support services as the diagnosis is often attributed to spiritual causes and this belief often interferes with treatment adherence [33]. Ascertaining and accurately documenting spiritual concerns and beliefs in relation to their diagnosis will assist HCPs to address the women’s spiritual concerns effectively [36]. One New Zealand study [49], that focused on improving the consistency and quality of spiritual care, reported that fostering inter-professional and patient collaboration can be useful to women with advanced breast cancer.

## Strengths and limitations

Due to the sampling methods used, transferability of the study findings should be undertaken with caution. In addition, the number of participating experts, particularly the key informants of breast cancer organisations, was limited. Most of the experts who participated in this study were aligned to only one of the two recruitment sites in this study. However, as this site is the second largest treating centre for women with breast cancer with multidisciplinary treating teams in Ghana, the experts’ breadth of experience is likely to be extensive. Furthermore, the categories generated from the interview data were consistent with the existing literature on the needs of this cohort.

## Conclusion

This study explored experts’ perspectives of the supportive care needs of women with advanced breast cancer in Ghana. As women continue to present in the advanced stages at healthcare facilities for breast cancer treatment, it is imperative to frequently assess their needs. The study findings highlight the need for more and improved evidence-based, tailored, supportive care interventions to be established for women with advanced breast cancer in Ghana particularly regarding information about their condition, treatments and related effects, as well as spiritual support and guidance due to the fatalistic beliefs they often associate with the condition. Additional research is needed to explore how multidisciplinary teams can work collaboratively to provide comprehensive support to the women in addressing their needs.

## Supplementary Information

**Additional file 1.** Interview guide.

## Data Availability

Data are available upon reasonable request from the corresponding author.

## Abbreviations

HCPs: Healthcare professionals
NGOs: Non-governmental organisations
COREQ: Consolidated criteria for reporting qualitative research
