# Supplementary material for: Healthcare providers’ perspectives of the supportive care needs of women with advanced breast cancer in Ghana
Source: BMC Womens Health. 2022 Aug 18;22:350. doi: 10.1186/s12905-022-01931-7 (PMC9389751; doi:10.1186/s12905-022-01931-7)
Supplement: Supplementary file 1 — Additional file 1. Interview guide. [file 12905_2022_1931_MOESM1_ESM.docx]

Additional file 1 Interview guide

| **Supportive Care Needs**  ***Informational needs***   1. What information do you think these women need once they know they have advanced disease? In what form, for how long and by who?   ***Psychological and Emotional needs***   1. In your experience, what are the psychological needs of women with ABC? 2. What are the emotional needs of the women in relation to their condition? 3. What sex-related problems do they encounter because of the disease?   ***Physical needs***   1. What physical needs do you think these women experience?   ***Practical needs***   1. What are the practical needs of these women? 2. What impact does the disease have on their usual daily activities, finances and employment?   ***Social needs***   1. What are the social needs of these women? 2. What impact does the disease have on the women’s relationships? 3. What difficulties have the women with ABC expressed in relation to talking with their families or friends about their diagnosis?   ***Spiritual needs***   1. What are the spiritual needs of these women?   **Health and Support Services Needs**   1. What support services are currently being provided to women with ABC to address their needs by your facility/organisation? |
| --- |
